# Machine learning algorithms for the prediction of adverse prognosis in patients undergoing peritoneal dialysis

**DOI:** 10.1186/s12911-023-02412-z

**Published:** 2024-01-02

**Authors:** Jie Yang, Jingfang Wan, Lei Feng, Shihui Hou, Kaizhen Yv, Liang Xu, Kehong Chen

**Affiliations:** 1 Department of Nephrology, Daping Hospital, Army Medical University, Chongqing, 400042 China; 2 Teaching Office, Medical Research Department, Army Special Medical Center, Chongqing, China; 3 Department of Medical Engineering, The Second Affiliated Hospital of the Army Medical University, Chongqing, 400037 China; 4 State Key Laboratory of Trauma, Burns and Combined Injury, Wound Trauma Medical Center, Army Medical University, Chongqing, China

**Keywords:** Machine learning, Peritoneal dialysis, Prediction model, Prognosis

## Abstract

**Background:**

An appropriate prediction model for adverse prognosis before peritoneal dialysis (PD) is lacking. Thus, we retrospectively analysed patients who underwent PD to construct a predictive model for adverse prognoses using machine learning (ML).

**Methods:**

A retrospective analysis was conducted on 873 patients who underwent PD from August 2007 to December 2020. A total of 824 patients who met the inclusion criteria were included in the analysis. Five commonly used ML algorithms were used for the initial model training. By using the area under the curve (AUC) and accuracy (ACC), we ranked the indicators with the highest impact and displayed them using the values of Shapley additive explanation (SHAP) version 0.41.0. The top 20 indicators were selected to build a compact model that is conducive to clinical application. All model-building steps were implemented in Python 3.8.3.

**Results:**

At the end of follow-up, 353 patients withdrew from PD (converted to haemodialysis or died), and 471 patients continued receiving PD. In the complete model, the categorical boosting classifier (CatBoost) model exhibited the strongest performance (AUC = 0.80, 95% confidence interval [CI] = 0.76–0.83; ACC: 0.78, 95% CI = 0.72–0.83) and was selected for subsequent analysis. We reconstructed a compression model by extracting 20 key features ranked by the SHAP values, and the CatBoost model still showed the strongest performance (AUC = 0.79, ACC = 0.74).

**Conclusions:**

The CatBoost model, which was built using the intelligent analysis technology of ML, demonstrated the best predictive performance. Therefore, our developed prediction model has potential value in patient screening before PD and hierarchical management after PD.

**Supplementary Information:**

The online version contains supplementary material available at 10.1186/s12911-023-02412-z.

## Background

Peritoneal dialysis (PD) is one of the main renal replacement treatments for end-stage renal disease (ESRD, also known as uraemia) [1]. The average number of new ESRD diagnoses worldwide is 144 individuals per million of the general population [2], of which approximately 11% receive PD [3]. The international PD guidelines recommend family-based renal replacement therapy owing to the prevalence of coronavirus 2019, and it has become the first choice for dialysis patients because of its simplicity and low cost [4]. However, factors such as peritonitis and peritoneal fibrosis lead to the failure of PD technology. The failure of PD technology limits its application, leading to patient withdrawal owing to the cost, and lowers patient survival rates, even leading to death [5, 6]. Early prediction may identify patients who are at high risk of PD technology failure in the short term and help determine whether to choose PD for renal replacement therapy.

With the exponential growth in healthcare data, machine learning (ML) is expected to provide more accurate and personalised services when processing large-scale medical data, predicting the development and prognosis of diseases, assisting doctors in formulating treatment plans, and identifying new disease risk factors and treatment methods. ML is also expected to promote the progress and development of medical science. An ML algorithm was used to evaluate the accuracy (ACC) of predicting cardiovascular events in asymptomatic populations by comparing random survival forests (an ML technique) with standard cardiovascular risk scores [7]. The prognostic factors affecting kidney transplant surgery cover multiple fields of surgery, immunology, epidemiology, and physiology; the large amount of data that is generated can precisely leverage the computational power of ML [8]. However, studies using ML algorithms for PD-related prognosis are limited.

ML technology was used to predict the prognosis, survival, and death risk factors of patients with PD and reported that deep neural networks demonstrated the best predictive performance (area under the curve [AUC]: 0.841) [9]. In patients with PD-associated peritonitis, traditional microbiology and molecular biology methods are considerably slow and have limited clinical applications. ML has demonstrated the power of using nonlinear methods to mine complex biomedical datasets to rapidly predict the fine reactivity and specificity of the human immune system and target antibiotic medication for early patient treatment [10]. Myopenia is associated with cardiovascular risk and mortality in patients with PD, and the ML model can effectively predict PD myopenia using simple clinical indicators [11].

However, we lack an appropriate prediction model for the adverse prognosis before PD; therefore, we constructed a prediction model for the adverse prognosis of PD using ML based on the data from our medical centre.

## Materials and methods

### Subjects

We retrospectively analysed 873 patients who underwent PD at our institution from August 2007 to December 2020. The inclusion criteria were 1) diagnosis of chronic renal failure and regular PD treatment for over 1 month and 2) age of 16 years or older. The exclusion criteria were 1) patients with acute kidney injuries, patients who received emergency PD, and patients in renal function recovery; 2) patients with incomplete baseline data; 3) patients who received kidney transplantation during follow-up; and 4) patients who stopped communicating with our medical centre. On the basis of these criteria, 824 patients were included in our subsequent analyses. This study was approved by the Medical Ethics Committee of Daping Hospital (YYLS2022-210), and all methods were carried out in accordance with relevant guidelines and regulations or declaration of Helsinki.

### Demographic and clinical information

All baseline data were collected before PD, and the variable collection period was one week before the start of PD. The baseline variable was the last value of the patient before the start of PD. The patient demographic data were as follows: age (years), sex (male/female), height (cm), weight (kg), body surface area (m^2^), body mass index (BMI) (kg/m^2^), Admitted_date (date of formal dwelling with peritoneal dialysate), marital status (unmarried, married, divorced, widowed), education level (primary school and below, junior high school, high school, college, undergraduate, master’s degree or above), ethnicity (Han, other ethnic minorities), smoking history (yes/no), history of alcohol consumption (yes/no), systolic blood pressure (SBP) (mmHg), diastolic blood pressure (DBP) (mmHg), heart rate (beats/min), urine volume (ml/24 h), primary disease, comorbidities, dialysis term (months), previous history of renal replacement therapy (including haemodialysis and kidney transplantation), and medication history. The laboratory data were as follows: haemoglobin (HGB), ferritin, serum iron, serum total iron binding capacity (TIBC), transferrin saturation, blood calcium, blood phosphorus, intact parathyroid hormone (iPTH), calcium–phosphorus product, alkaline phosphatase, serum albumin, prealbumin, blood sodium, blood potassium, blood chlorine, carbon dioxide binding capacity, creatinine, urea nitrogen, uric acid β2 microglobulin, estimated glomerular filtration rate (eGFR), total cholesterol (TC), triglyceride, low-density lipoprotein cholesterol (LDL-c), high-density lipoprotein cholesterol (HDL-c), TIBC, serum ferritin (SF), fasting blood glucose (FBG), glycosylated HGB, β-type natriuretic peptide, troponin, creatine kinase (CK), CK myoglobin (CKMB), C-reactive protein (CRP), vitamin D (Vd), erythrocyte sedimentation rate (ESR), hepatitis B surface antigen, hepatitis C antigen/antibody, syphilis antibody, and human immunodeficiency virus antibody.

The imaging techniques were as follows: echocardiography (left ventricular end-diastolic diameter, interventricular septum thickness, left ventricular posterior wall thickness, and calculation of the left ventricular mass and left ventricular mass index), and carotid artery colour Doppler ultrasound (the presence of plaque formation).

The adverse prognosis was defined as the withdrawal from PD or all-cause mortality within 24 months of PD initiation. The patients were divided into PD withdrawal and PD continuation groups according to whether an adverse prognostic event occurred. If a patient withdrew from PD during follow-up, the time and reason for withdrawal (peritonitis, insufficient dialysis, ultrafiltration failure, thoracoabdominal fistula, catheter dysfunction, patient requirements, and other causes) were recorded. If a patient died, the dialysis duration and cause of death (cardiovascular death, other causes) were recorded. The study was terminated on 31 December 2020.

### Statistical analysis

The measurement data are expressed as the mean ± standard deviation, and the counting data are expressed as a percentage. The measurement data between the groups were compared using the t-test, and the two group rates were compared using the chi-square test. The data were processed using the Statistical Package for the Social Sciences version 20.0. ML methods were used to construct a predictive model for the adverse prognosis in patients with PD. During the model construction, the enrolled patients were randomly divided into two groups at a ratio of 7:3. The larger group was the training subset for ML, and the smaller group was the testing subset for model testing. A small number of missing continuous variables were supplemented using the median method, and the categorical variables were supplemented using the 0-value method.

The following five commonly used ML algorithms were used for the initial model training: categorical boosting classifier (CatBoost) version 1.0.6, logistic regression (LR) version 1.0.2, light gradient boosting (LGB) version 3.2.1, gradient boosting (GBT) version 1.0.2, and random forest (RFL) version 1.0.2. LR is a type of generalised linear regression. The advantage is that the rate function is derivable to any order and has good mathematical properties. Many existing numerical optimisation algorithms can be used to find the optimal solution. The disadvantage is that LR cannot be used to solve nonlinear problems and cannot address the problem of data imbalance. In ML, the goal is to train the model successfully with multiple learning algorithms. Boosting is a method that is often used in practice and is not built in parallel but sequentially. The weak algorithm first trains the model and then reassembles the model according to the training results to improve the learning rate of the model. GBT is the most basic boosting model algorithm, which has no role in optimising complex data types and missing data. LGB is a highly effective way to reduce errors and improve ACC and speed; however, it does not support strings and requires a special algorithm to split the classified data. LGB performs better than CatBoost on large datasets and high-dimensional data, whereas CatBoost is better than LGB at handling category features and missing values. CatBoost is a symmetric decision tree–based learner, which relies on the GBT framework and a small number of parameters, supports categorical variables, and has high ACC. The optimisation algorithm formula of CatBoost is as follows:

$$\widehat x_k^i=\frac{\sum_{{xj\in D}_k}\mathbbm{1}_{x_j^i=x_k^i}\cdot y_j+ap}{\sum_{{xj\in D}_k}\mathbbm{1}_{x_j^i=x_k^i}+a},$$

where D is the set of all data available to train and evaluate our ensemble. CatBoost chooses the data to use for fitting by placing an arbitrary order on the elements of D with a random permutation σ. Let σ(k) be the kth element of D under σ, and Dk = {x_1_, x_2_, …, x_k−1_}, as ordered by the random permutation σ. Another concept for understanding how CatBoost encodes the values of categorical variables is the indicator function $\mathbbm{1}_{a=b}$, which is a function of one variable that has the value of one when a = b and zero otherwise. This indicator function plays an important role in the formula applied by CatBoost to map the values of a categorical feature to a numerical value. Specifically, this formula involves the indicator function $\mathbbm{1}_{{x}_{j}^{i}={x}_{k}^{i}}$, which takes the value one when the ith component of input vector x_j_ of CatBoost is equal to the ith component of input vector x_k_. p is the added prior term, and a is usually a weight coefficient greater than zero. For binary classification problems, the prior term is the prior probability of the positive example. These concepts enable us to define the formula for the encoded value ${\widehat{x}}_{k}^{i}$.

The AUC, ACC, F1 score, and precision recall curve were used as the primary evaluation indices to select the optimal model for further optimisation. We obtained the ranking of the indicators that had the most significant impact on the model and displayed them using the Shapley additive explanation (SHAP, version 0.41.0) values, from which the top 20 indicators were selected to build a compact model conducive to clinical application. All the model-building steps were implemented in Python (version 3.8.3).

## Results

According to the inclusion criteria, nine patients with a dialysis duration of less than one month at the time of withdrawal were excluded. During follow-up, 31 patients underwent kidney transplantation, and nine were lost. A total of 824 patients were included in the analysis at the end of follow-up, 353 patients withdrew from PD (converted to haemodialysis or died), and 471 patients continued receiving PD.

Our cohort included 481 men and 343 women with an average age of 47.82 ± 15.45 years, and most were married (91.6%) and of the Han ethnicity (94.7%). The education level was mainly middle school (37.0%), with smokers accounting for 24.7% and alcohol consumption accounting for 15.6%. The three primary causes of chronic renal failure were chronic glomerulonephritis in 479 patients (58.1%), diabetic nephropathy in 112 patients (13.5%), and hypertensive renal injury in 21 patients (2.5%). The most common complications were hypertension (*n* = 454, 55.0%). Significant differences in age, education level, urinary output, history of kidney transplantation, primary renal disease, comorbidity, and history of medication were identified between the two groups. Demographic data are presented in Table 1.

**Table 1:** Patient demographics and clinical characteristics

Characteristics	Total (*N*: 824)	PD continuation group (*N*: 471)	PD withdrawal group (*N*: 353)	*P*
Age (years)	47.82 ± 15.45	45.36 ± 14.46	51.11 ± 16.12	< 0.001
Sex (male)/N (%)	481 (58.3%)	263 (55.8%)	218 (61.7%)	0.088
BMI, kg/m^2^	22.98 ± 3.62	22.89 ± 3.74	23.1 ± 3.46	0.421
Body surface area (m^2^)	1.61 ± 0.19	1.6 ± 0.19	1.62 ± 0.18	0.350
Marital status				0.216
Unmarried	62 (7.5%)	42 (8.9%)	20 (5.6%)	
Married	755 (91.6%)	426 (90.4%)	329 (93.2%)	
Divorced	6 (0.7%)	3 (0.6%)	3 (0.8%)	
Widowed	1 (0.1%)	0	1 (0.2%)	
Education level				0.004
< 6 years	185 (22.4%)	69 (14.6%)	116 (32.8%)	
6–9 years	305 (37.0%)	177 (37.5%)	128 (36.2%)	
9–12 years	151 (18.3%)	97 (20.8%)	54 (15.2%)	
12–17 years	81 (9.8%)	54 (11.4%)	27 (7.6%)	
> 17 years	3 (0.3%)	2 (0.4%)	1 (0.2%)	
Ethnicity, Han/N (%)	781 (94.7%)	449 (95.3%)	332 (94.0%)	0.414
Smoking history/N (%)	204 (24.7%)	115 (24.4%)	89 (25.2%)	0.793
Drinking History/N (%)	129 (15.6%)	73 (15.4%)	56 (15.8%)	0.887
Systolic blood pressure (mmHg)	150.47 ± 26.13	150.14 ± 25.34	150.92 ± 27.19	0.672
Diastolic blood pressure (mmHg)	86.23 ± 18.79	87.12 ± 18.08	85.03 ± 19.67	0.115
Heart rate (bpm)	86.28 ± 13.86	87.09 ± 14.28	85.19 ± 13.22	0.052
Urinary output (ml/24 h)	1013.71 ± 473.18	1048.09 ± 448.97	967.84 ± 500.65	0.016
History of kidney transplantation/N (%)	8 (0.9%)	1 (0.2%)	7 (1.9%)	0.027
History of haemodialysis/N (%)	36 (4.3%)	17 (3.6%)	19 (5.3%)	0.218
Primary renal disease				0.001
Glomerulonephritis	479 (58.1%)	298 (63.2%)	181 (51.2%)	
Diabetic kidney disease	112 (13.5%)	42 (8.9%)	70 (19.8%)	
Hypertension	21 (2.5%)	9 (1.9%)	12 (3.3%)	
Obstructive nephropathy	10 (1.2%)	6 (1.2%)	4 (1.1%)	
Lupus nephritis	8 (0.9%)	3 (0.6%)	5 (1.4%)	
Cystic kidney disease	5 (0.6%)	3 (0.6%)	2 (0.5%)	
Renal vasculitis	5 (0.6%)	3 (0.6%)	2 (0.5%)	
Others	28 (3.3%)	13 (2.7%)	15 (4.2%)	
Unknown	156 (18.9%)	94 (19.9%)	62 (17.5%)	
Comorbidity/N (%)				
Diabetes mellitus	135 (16.3%)	58 (12.3%)	77 (21.8%)	< 0.001
Hypertension	454 (55.0%)	257 (54.5%)	197 (55.8%)	0.723
Coronary heart disease or myocardial infarction	48 (5.8%)	25 (5.3%)	23 (6.5%)	0.464
Congestive heart failure	17 (2.0%)	7 (1.4%)	10 (2.8%)	0.178
Cardiac arrhythmias	14 (1.6%)	9 (1.9%)	5 (1.4%)	0.587
History of stroke or cerebral vascular diseases	25 (3.0%)	8 (1.6%)	17 (4.8%)	0.010
Malignancies	7 (0.8%)	3 (0.6%)	4 (1.1%)	0.442
Peripheral arterial disease	8 (0.9%)	3 (0.6%)	5 (1.4%)	0.259
Urology procedures	12 (1.4%)	6 (1.2%)	5 (1.6%)	0.860
History of medication				
ARB	376 (45.6%)	220 (46.7%)	156 (44.1%)	0.473
ACEI	40 (4.8%)	11 (2.3%)	29 (8.2%)	< 0.001
CCB	654 (79.3%)	369 (78.3%)	285 (80.7%)	0.401
Diuretic	160 (19.4%)	99 (21.0%)	61 (17.2%)	0.179
EPO	604 (73.3%)	368 (78.1%)	236 (66.8%)	< 0.001
Uric acid-lowering medications	110 (13.3%)	87 (18.4%)	23 (6.5%)	< 0.001
Iron	144 (17.4%)	74 (15.7%)	70 (19.8%)	0.123
β-receptor blockade	185 (22.4%)	109 (23.1%)	76 (21.5%)	0.583
α-receptor blockade	187 (22.6%)	95 (20.1%)	92 (26.0%)	0.046
α/β-receptor blockade	60 (7.2%)	25 (5.3%)	35 (9.9%)	0.012
α-ketoacids	226 (27.4%)	112 (23.7%)	114 (32.2%)	0.007
Antidiabetic agents	115 (13.9%)	49 (10.4%)	66 (18.6%)	0.001
Lipid-lowering medications	115 (13.9%)	68 (14.4%)	42 (11.8%)	0.289
Sleep aids	11 (1.3%)	6 (1.2%)	5 (1.4%)	0.109
Glucocorticoids	16 (1.9%)	6 (1.2%)	10 (2.8%)	0.154
Immunosuppressive agents	10 (1.2%)	3 (0.6%)	7 (1.9%)	0.081
Calcimimetic agents	3 (0.3%)	2 (0.4%)	1 (0.2%)	1.000

*BMI* Body mass index, *PD* Peritoneal dialysis, *ARB* Angiotensin receptor blockers, *ACEI* Angiotensin-converting enzyme inhibitors, *CCB* Calcium channel blockers, *EPO* Erythropoietin

The PD withdrawal group had higher levels of ferritin, blood calcium, alkaline phosphatase, eGFR, LDL-c, FBG, glycated HGB, CRP, cardiac ejection fraction, blood phosphorus, iPTH, serum albumin, creatinine, urea nitrogen, and uric acid. β2 microglobulin was lower in the PD continuation group, and we observed no statistically significant differences in the other indicators (Table 2).

**Table 2:** Patient baseline laboratory data for peritoneal dialysis

Characteristics	Total	PD continuation group (*N*: 471)	PD withdrawal group (*N*: 353)	t/ χ^2^	*p*
	(*N*: 824)				
Haptoglobin	81.23 ± 18.41	81.02 ± 19.05	81.5 ± 17.55	− 0.376	0.707
Ferritin	290.04 ± 289.57	270.31 ± 263.08	316.78 ± 320.55	− 2.06	0.040
Serum iron	12.67 ± 7.58	12.72 ± 7.61	12.6 ± 7.55	0.210	0.834
Total iron binding capacity	44.17 ± 9.19	43.84 ± 8.82	44.66 ± 9.73	− 1.189	0.235
Transferrin saturation	29.35 ± 17.16	29.69 ± 16.89	28.85 ± 17.58	0.651	0.516
Serum calcium	1.93 ± 0.28	1.91 ± 0.29	1.95 ± 0.27	− 2.188	0.029
Serum phosphorus	2.03 ± 0.62	2.1 ± 0.62	1.94 ± 0.6	3.699	< 0.001
Intact parathormone	396.61 ± 257.89	420.99 ± 264.14	359.5 ± 243.89	3.190	0.001
Calcium-phosphorus product	4.15 ± 1.24	4.2 ± 1.2	4.09 ± 1.29	1.243	0.214
Alkaline phosphatase	92.63 ± 45.68	89.52 ± 42.86	97.27 ± 49.33	− 2.149	0.032
Serum albumin	32.84 ± 5.61	33.79 ± 5.21	31.58 ± 5.88	5.707	< 0.001
Prealbumin	302.51 ± 88.02	309.74 ± 82.38	291.27 ± 95.2	2.692	0.007
Serum sodium	138.31 ± 4.01	138.5 ± 3.95	138.05 ± 4.07	1.615	0.107
Serum potassium	4.59 ± 0.87	4.59 ± 0.82	4.59 ± 0.94	− 0.028	0.978
Serum chloremia	104.88 ± 5.81	105.21 ± 5.74	104.44 ± 5.89	1.879	0.061
Carbon dioxide combining power	18.84 ± 4.67	18.72 ± 4.65	19.01 ± 4.7	− 0.882	0.378
Serum creatinine	891.45 ± 358.22	920.84 ± 359.19	852.24 ± 353.64	2.731	0.006
Serum urea nitrogen	28.48 ± 12.96	29.85 ± 13.92	26.66 ± 11.34	3.513	< 0.001
Uric acid	492.67 ± 139.6	502.99 ± 136.61	478.93 ± 142.52	2.455	0.014
β2 microglobulin	21.55 ± 8.74	22.67 ± 8.58	19.89 ± 8.72	3.669	< 0.001
eGFR	5.68 ± 2.31	5.47 ± 2.13	5.96 ± 2.5	− 3.036	0.002
Total cholesterol	4.15 ± 1.24	4.13 ± 1.25	4.18 ± 1.23	− 0.571	0.568
Triglyceride	1.54 ± 1.07	1.58 ± 1.08	1.49 ± 1.04	1.057	0.291
Lower blood lipids	2.52 ± 0.88	2.59 ± 0.87	2.43 ± 0.89	2.480	0.013
High-density lipoprotein	1.17 ± 0.52	1.16 ± 0.49	1.2 ± 0.56	− 0.942	0.347
Fasting blood glucose	5.07 ± 1.97	4.88 ± 1.46	5.33 ± 2.49	− 2.910	0.004
Glycosylated haemoglobin A1c	5.33 ± 0.9	5.21 ± 0.88	5.5 ± 0.9	− 3.168	0.002
B-type Natriuretic Peptide	2062.5 ± 4874.21	1834.29 ± 4537.45	2500.66 ± 5485.42	− 0.783	0.435
Troponin	0.07 ± 0.09	0.07 ± 0.08	0.08 ± 0.1	− 1.245	0.214
Creatine kinase Myoglobin	3.67 ± 6.79	3.57 ± 6.97	3.86 ± 6.44	− 0.466	0.642
Creatine kinase	279.14 ± 342.13	279.44 ± 337.61	278.58 ± 351.5	0.028	0.978
C-reactive protein	12.36 ± 27.05	9.25 ± 21.24	17.67 ± 34.2	− 3.367	0.001
Erythrocyte sedimentation rate	56.2 ± 38.08	54.69 ± 35.74	59.29 ± 42.46	− 0.994	0.321
Hepatitis B surface antigen	70 (8.4%)	40 (8.4%)	30 (8.4%)	< 0.001	0.998
Hepatitis C antigen	11 (1.3%)	4 (0.8%)	7 (1.9%)	1.202	0.273
Syphilis antibody	2 (0.2%)	1 (0.2%)	1 (0.2%)	< 0.001	1.000
Left ventricular end-diastolic dimensions	4.93 ± 3.76	4.81 ± 3.01	5.26 ± 5.38	− 0.879	0.380
Interventricular septal thickness	1.19 ± 0.76	1.16 ± 0.6	1.29 ± 1.11	− 1.256	0.210
Left ventricular posterior wall thickness	1.07 ± 0.16	1.07 ± 0.16	1.08 ± 0.17	− 0.516	0.606
Left ventricular mass	217.97 ± 70.66	215.91 ± 70.97	226.39 ± 69.49	−0.921	0.358
Left ventricular mass index	133.33 ± 40.04	132.18 ± 40.18	138.03 ± 39.53	− 0.906	0.366
Left ventricular ejection fraction	64.02 ± 7.07	64.87 ± 6.63	61.67 ± 7.76	3.331	0.001
Carotid artery plaque/N (%)	28 (3.3%)	16 (3.3%)	12 (3.3%)	< 0.001	0.998

*eGFR* Estimated glomerular filtration rate, *PD* Peritoneal dialysis

### Comparison of the five complete models

**Fig. 1:**
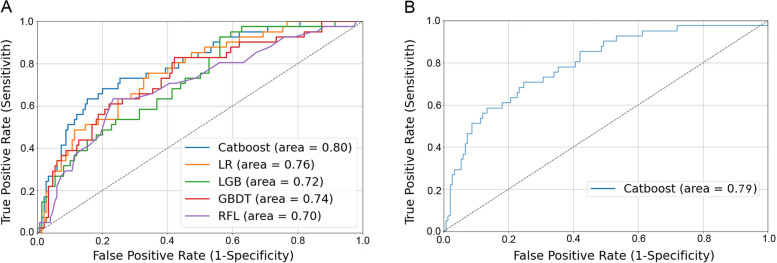
The ROC curves of the models. **A** The complete model ROC curves of five algorithms. The CatBoost algorithm had the highest AUC of 0.80. **B** The compact model ROC curve of the optimal algorithm. The algorithm with the best performance in the complete model was adjusted, and the top 20 variables with the strongest correlation were selected to create a compact model with an AUC of 0.79. ROC, receiver operating characteristic; AUC, area under the curve

The performances of the different models are listed in Table 3. The CatBoost algorithm exhibited an excellent AUC of 0.80 (95% confidence interval [CI]: 0.76–0.83) and an ACC of 0.78 (95% CI: 0.72–0.83) values. The prediction performance of the traditional LR method was acceptable, with an AUC of 0.76 (95% CI: 0.73–0.80) and an ACC of 0.71 (95% CI: 0.64–0.77). The performances of the other three ensemble learning algorithms, LGB (AUC: 0.72; ACC: 0.74), GBT (AUC: 0.72; ACC: 0.76), and RFL (AUC: 0.72; ACC: 0.65), were relatively poor. The receiver operating characteristic (ROC) curve of the complete model is displayed in Fig. 1a. The CatBoost model exhibited the strongest performance and was selected for subsequent analysis.

**Table 3:** Basic performance indicators of the five complete models

Model name	AUC (CI)	ACC (CI)	F1 score (CI)	PRC (CI)
Cat Boost Classifier	0.8	0.78	0.57	0.52
	[0.76, 0.83]	[0.72, 0.83]	[0.50, 0.64]	[0.45, 0.60]
Logistic Regression	0.76	0.71	0.48	0.45
	[0.73, 0.80]	[0.64, 0.77]	[0.44, 0.52]	[0.39, 0.52]
Light Gradient Boosting	0.72	0.74	0.42	0.44
	[0.68, 0.77]	[0.68, 0.81]	[0.35, 0.48]	[0.36, 0.50]
Gradient Boosting	0.72	0.76	0.41	0.35
	[0.70, 0.79]	[0.70, 0.82]	[0.34, 0.51]	[0.31, 0.43]
Random Forest	0.72	0.65	0.41	0.41
	[0.62, 0.75]	[0.58, 0.72]	[0.37, 0.45]	[0.34, 0.48]

*ACC* Accuracy, *AUC* Area under the curve, *CI* Confidence interval, *F1* F1 score, *PRC* Precision recall curve, *CI* Confidence interval

### Key features and compact model

**Fig. 2:**
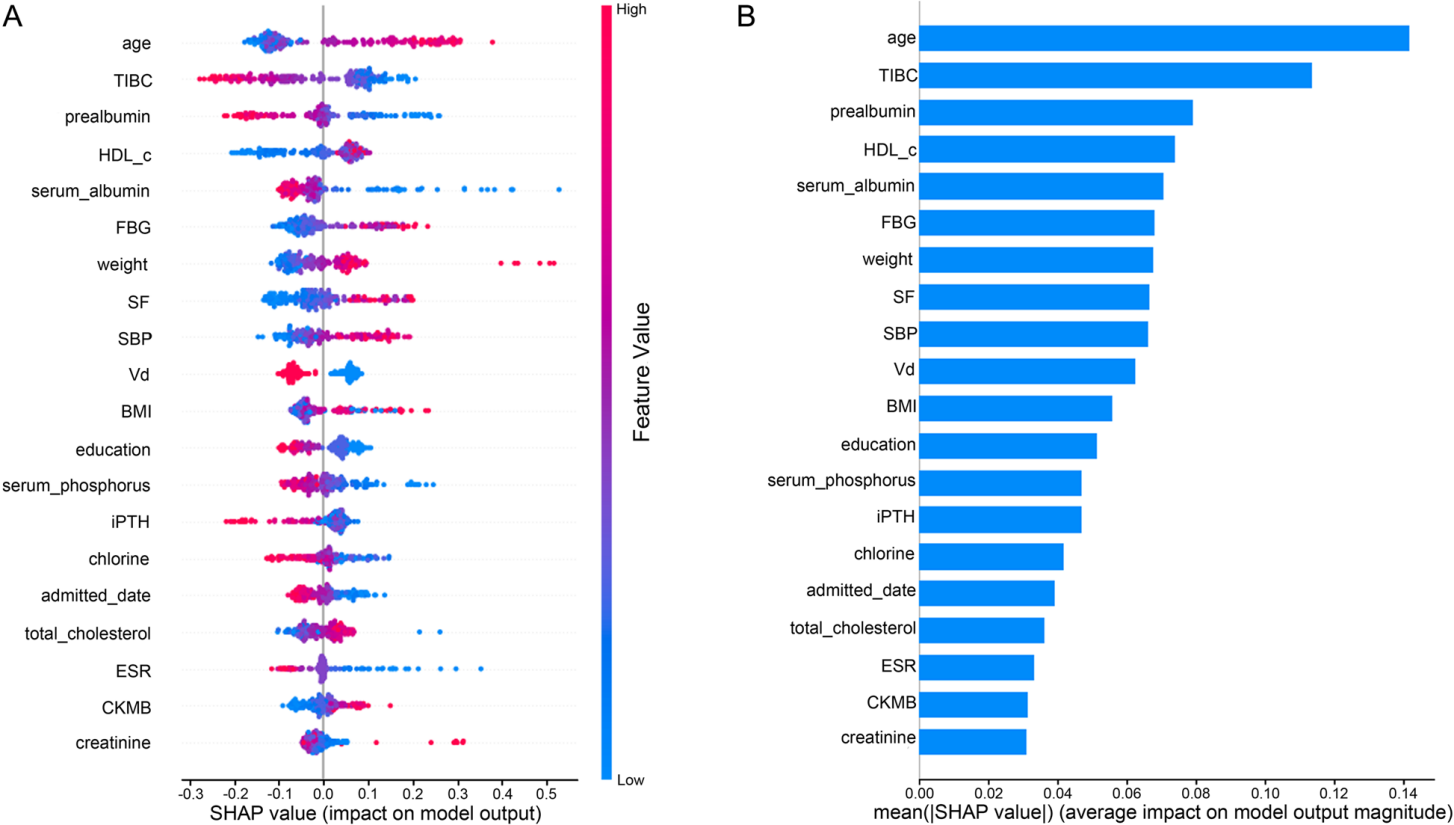
The SHAP values of the Catboost model. **A** The variables with the strongest correlation in the prediction model were ranked, and the top 20 were obtained. **B** The SHAP value of these variables. SHAP, Shapley additive explanation

After excluding 187 individuals because of missing data on covariates or the predictor variables of interest, 637 patients were included in the final model construction. The calculated SHAP values summarised the ranking of the features that had the strongest influence on the prediction results of the complete model. The feature names and the extent of their influence are presented in Fig. 2. Among the demographic characteristics, age, weight, BMI, and education level were selected as significant predictors of adverse PD prognosis. Iron metabolism was closely related to haematopoiesis, among which TIBC and SF were key predictors. Prealbumin and serum albumin levels, which were closely related to liver synthesis, also played important roles. There were also prominent roles for HDL-c, FBG, and TC in glycolipid metabolism. In addition, calcium and phosphorus metabolism (Vd, serum phosphorus, and iPTH), cardiovascular function (SBP, CKMB), ESR, and creatinine had some predictive effects.

We reconstructed a compression model by extracting 20 key features ranked by the SHAP values. This simplified version of the model (AUC: 0.79; ACC: 0.74) was slightly weaker in performance than the full model but was more conducive to clinical application and data collection (Fig. 1b). The complement model had a maximum Youden index of 0.48, which gives a sensitivity of 0.68 and a specificity of 0.80. The maximum Youden index of the compact model was 0.46, and the sensitivity and specificity were 0.71 and 0.75, respectively. The specific data parameters are listed in Table 4.

**Table 4:** Performance indicators of the final models

Model	Performance				
	ACC	AUC	Youden	Sensitivity	Specificity
Full	0.78 [0.72, 0.83]	0.80 [0.76, 0.83]	0.48	0.68 [0.53, 0.80]	0.80 [0.73, 0.86]
Compact	0.74 [0.68, 0.80]	0.79 [0.75, 0.84]	0.46	0.71 [0.56, 0.82]	0.75 [0.67, 0.81]

*ACC* Accuracy, *AUC* Area under the curve

### Model explanation

**Fig. 3:**
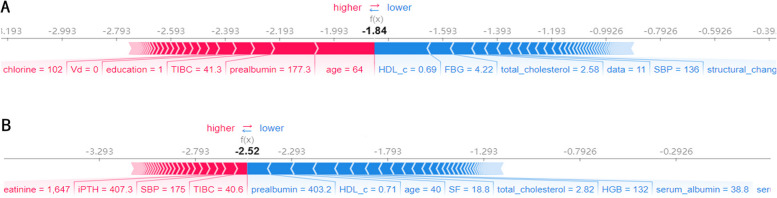
Two examples of model interpretation. **A** A patient who was predicted to be unfit for PD failed after a short period of PD. **B** A patient predicted to be suitable for PD succeeded for over two years and continued PD for five years

The summary plot of the SHAP values in Fig. 2 provides an overview of the impact of the features of the final model. Figure 3 illustrates two specific forecasting examples. The blue bars represent protective factors, where longer bars indicate that PD was less likely to fail. The red bars represent risk factors and indicate the opposite effect. As depicted in Fig. 3a, a 64-year-old patient with poor education, low TIBC, and prealbumin levels was suspected of having reduced hepatic compensatory function. The final model predicted that his PD would fail, and he became hospitalised with infectious peritonitis after 7.1 months of dialysis. For another 40-year-old patient, HGB, prealbumin, and albumin levels all appeared normal, indicating a strong compensatory capacity (Fig. 3b). The model predicted that the patient was suitable for PD, and the patient continued PD after the follow-up period for over five years.

## Discussion

PD-associated peritonitis is one of the leading causes of PD withdrawal and death [12, 13]. ML algorithms are becoming increasingly popular in medical research and can be applied to disease screening, diagnosis, and prognosis. We used ML intelligent analysis technology to construct a predictive model for the adverse prognosis of PD and demonstrated that age, body weight, and albumin levels are important predictive factors for the adverse prognosis of PD. We developed five predictive models; in the complete model, the calculated SHAP values summarised the strongest predictive indicators and sorted and extracted the 20 key features to reconstruct the model. Collectively, our findings suggested that the CatBoost model demonstrated the strongest performance.

We ranked the factors closely related to the adverse prognosis of patients by the SHAP values, with the top 20 key factors including age, body weight, albumin, and blood lipids. The meta-analysis revealed that age is a risk factor for all-cause cardiovascular death in dialysis patients [14]. In this study, we observed that the age of patients in the PD continuation group was significantly lower than that in the adverse prognosis group (45.36 vs 51.11 years, *P* < 0.001). In the complete model, the calculated SHAP values confirmed that age had the strongest impact on predicting an adverse prognosis for patients with PD. In addition, body weight and BMI were critical predictive factors for adverse PD prognosis, with higher BMI leading to higher hospitalisation rates for peritonitis [15]. In the general population, obesity is associated with increased cardiovascular risk and reduced survival, but the “obesity paradox” in ESRD has always been controversial [16, 17]. Our study suggests that increased body weight and BMI correlate with a lower risk of adverse PD prognosis. The nutritional indicators include body weight, as well as albumin and blood lipids. A positive correlation between nutritional status and dialysis duration has been reported in patients with PD because a nutritious diet reduces the incidence of complications such as peritonitis [12].

Education level was also considered a vital predictor of adverse PD prognosis, and multiple studies have demonstrated that [18, 19] patients with lower education levels experience increased peritonitis and technical failure than those with higher education levels. The potential reason may be that patients with lower education levels have lower incomes and poor compliance, which affects their access to timely healthcare, medication, and treatment.

The high prevalence of cardiovascular diseases in patients with PD is related to uremic toxins, inflammation (ESR), and disorders in bone mineral metabolism (Vd, serum phosphorus, and iPTH) [20]. Similarly, we observed Vd, serum phosphorus, iPTH, ESR, creatinine, and cardiovascular disease to be associated with adverse PD prognosis in patients. Furthermore, we observed that TIBC and SF are critical predictive factors for adverse PD prognosis and that higher amounts of iron increase the risk of QT dispersion [21]. Functional iron deficiency is an independent risk factor for all-cause death in patients with PD. Consistent with our research findings, patients with PD with high iron levels have a four-fold higher risk of all-cause cardiovascular death [22]. The effect of the COVID-19 pandemic on patients with PD is still being debated, and certain authors argue that COVID-19 has no effect on the survival of patients with PD [23]. However, other researchers state that the COVID-19 pandemic led to increased death [24, 25]. During the severe period of the COVID-19 pandemic, the mortality rate of patients with PD at our centre was 2.64%, whereas no significant changes were observed in the mortality rate of patients with PD (5.13%) over the same period. We believe that the COVID-19 pandemic had no significant effect the results of the model.

ML is an interdisciplinary field of mathematics and statistics [26] that involves fitting predictive models to data for information grouping. We assumed that ML methods could predict the adverse prognosis of patients before starting PD, recommended the most favourable dialysis method, and provided timely medical intervention, which improved patient prognosis and reduced medical costs.

CatBoost is the third Gradient-Boosted Decision Tree (GBDT)–based improved algorithm after XGBoost and LightGBM [27]. Launched by Yandex Company in Russia in 2018 and is open source. It uses gradient lifting on the decision tree and can be easily integrated into deep-learning frameworks. Based on the GBDT framework, which has fewer parameters, CatBoost supports categorical variables with high ACC and can efficiently and reasonably process t-algorithms. CatBoost has been extensively studied in the prediction of skin sensitisation [28], depression occurrence [29], pregnancy diabetes management [30], and transplanted kidney function [8], and it exhibits good predictive performance. Owing to numerous factors that affect an adverse PD prognosis and considering the clinical applications, we reconstructed a compression model by extracting 20 key features ranked by the SHAP values. This simplified version of the model was slightly weaker in performance than the full model but was more conducive to clinical application and data collection. Before a patient starts PD, the CatBoost model can be used to predict whether the patient is suitable for PD treatment and whether PD-related peritonitis may occur. On the basis of the prediction, the most optimal dialysis plan can be selected for the patient allowing early intervention.

Our study had several limitations. First, this was a single-centre retrospective study, and we could not evaluate whether the external cohort population exhibited the same pattern. Second, this study used the median of missing values, which inevitably led to bias. Third, the number of cases was relatively small, and the model construction lacked cross-validation and external validation, all of which affected the ability to generalise the model. A multicenter joint study is needed to validate the model. Finally, only patients with PD were included in the study. Therefore, the model might be strongly biased if it is used for patients with chronic kidney disease for the selection of the best renal replacement therapy.

## Conclusions

Collectively, the CatBoost model built using the intelligent analysis technology of ML demonstrated the best predictive performance (AUC: 0.79; ACC: 0.74). Thus, the model has potential value in patient screening before PD and hierarchical management after PD.

### Supplementary Information

**Additional file 1: Supplemental Figure 1. **Prediction accuracy of the models with “dead patients” and “PD withdrawal” within 24 months. Patient status after 24 months in the training subset. B. Patient status after 24 months in the test subset. C. AUC of the training model when dead patients were excluded. D. Confusion matrix of the training model when dead patients were excluded. E. AUC with patient death as the predictive outcome. F. Confusion matrix with patient death as the predictive outcome. **Supplemental Figure 2. **Stack of distributions with missing variables. m: number of missing variables.

**Additional file 2: Supplemental Table 1. **The SHAP value of the top 20 variables.

## Data Availability

The datasets used and/or analysed in the study are available from the corresponding author on reasonable request.

## Abbreviations

PD: Peritoneal dialysis
ML: Machine learning
SHAP: Shapley additive explanation
ESRD: End-stage renal disease
SBP: Systolic blood pressure
DBP: Diastolic blood pressure
HGB: Haemoglobin
iPTH: Intact parathyroid hormone
eGFR: Estimated glomerular filtration rate
LDL-c: Low-density lipoprotein cholesterol
HDL-c: High-density lipoprotein cholesterol
CK: Creatine kinase
CKMB: Creatine kinase myoglobin
CRP: C-reactive protein
TIBC: Total iron binding capacity
SF: Serum ferritin
FBG: Fasting blood glucose
ESR: Erythrocyte sedimentation rate
LR: Logistic regression
LGB: Light gradient boosting
GBT: Gradient boosting
RFL: Random forest
AUC: Area under the curve
ACC: Accuracy
ROC: Receiver operating characteristic
F1: F1 score
PRC: Precision recall curve
CI: Confidence interval
