# Supplementary material for: Machine learning algorithms for the prediction of adverse prognosis in patients undergoing peritoneal dialysis
Source: BMC Med Inform Decis Mak. 2024 Jan 2;24:8. doi: 10.1186/s12911-023-02412-z (PMC10763100; doi:10.1186/s12911-023-02412-z)
Supplement: Supplementary file 1 — Additional file 1: Supplemental Figure 1. Prediction accuracy of the models with “dead patients” and “PD withdrawal” within 24 months. Patient status after 24 months in the training subset. B. Patient status after 24 months in the test subset. C. AUC of the training model when dead patients were excluded. D. Confusion matrix of the training model when dead patients were excluded. E. AUC with patient death as the predictive outcome. F. Confusion matrix with patient death as the predictive outcome. Supplemental Figure 2. Stack of distributions with missing variables. m: number of missing variables. [file 12911_2023_2412_MOESM1_ESM.docx]

**Figure legends**

**Supplemental Figure 1.** Prediction accuracy of the models with “dead patients” and “PD withdrawal” within 24 months

1. Patient status after 24 months in the training subset. **B.** Patient status after 24 months in the test subset. **C.** AUC of the training model when dead patients were excluded. **D.** Confusion matrix of the training model when dead patients were excluded. **E.** AUC with patient death as the predictive outcome. **F.** Confusion matrix with patient death as the predictive outcome.

**Supplemental Figure 2.** Stack of distributions with missing variables. m: number of missing variables.


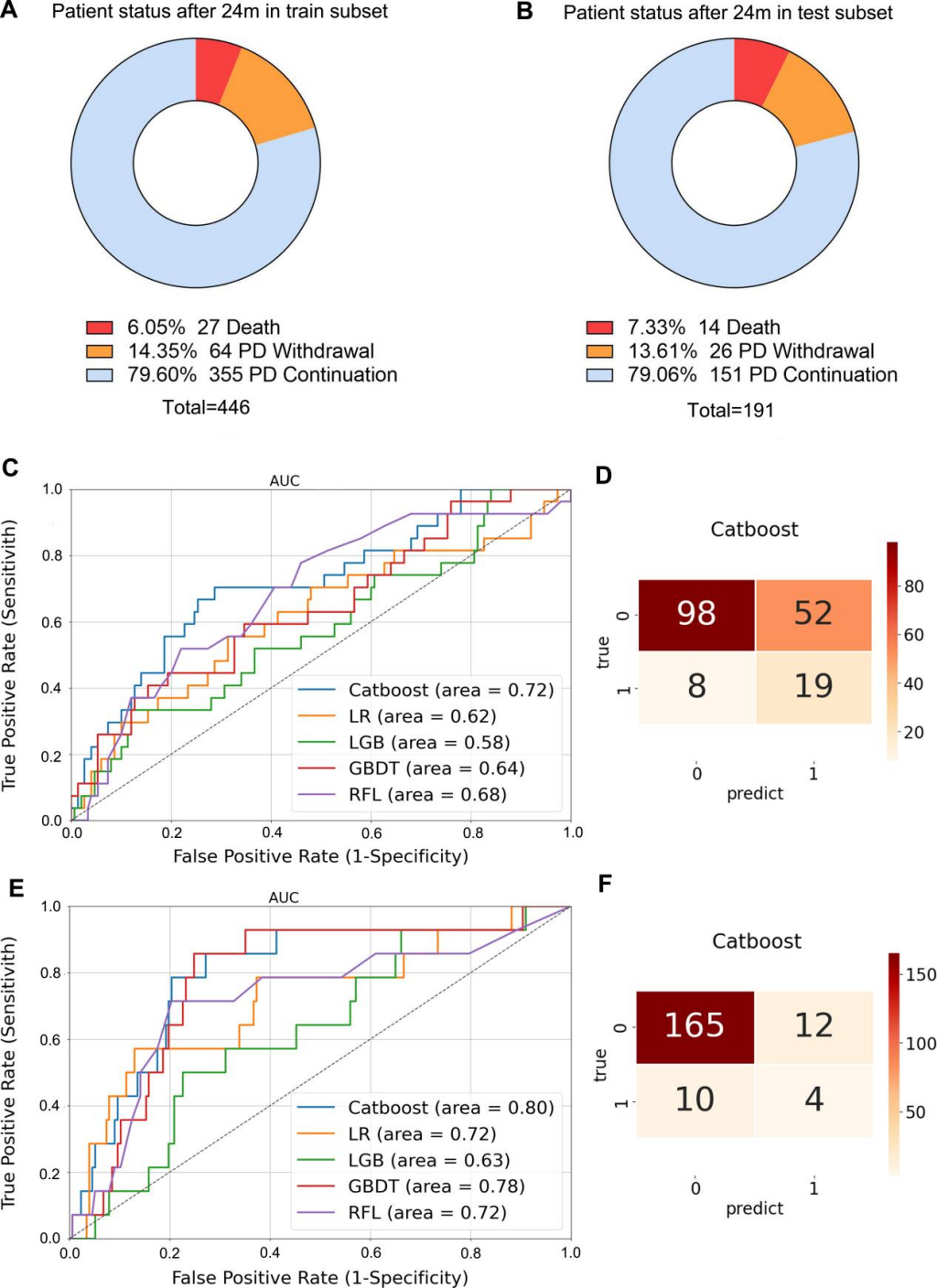


**Supplemental Figure 1**


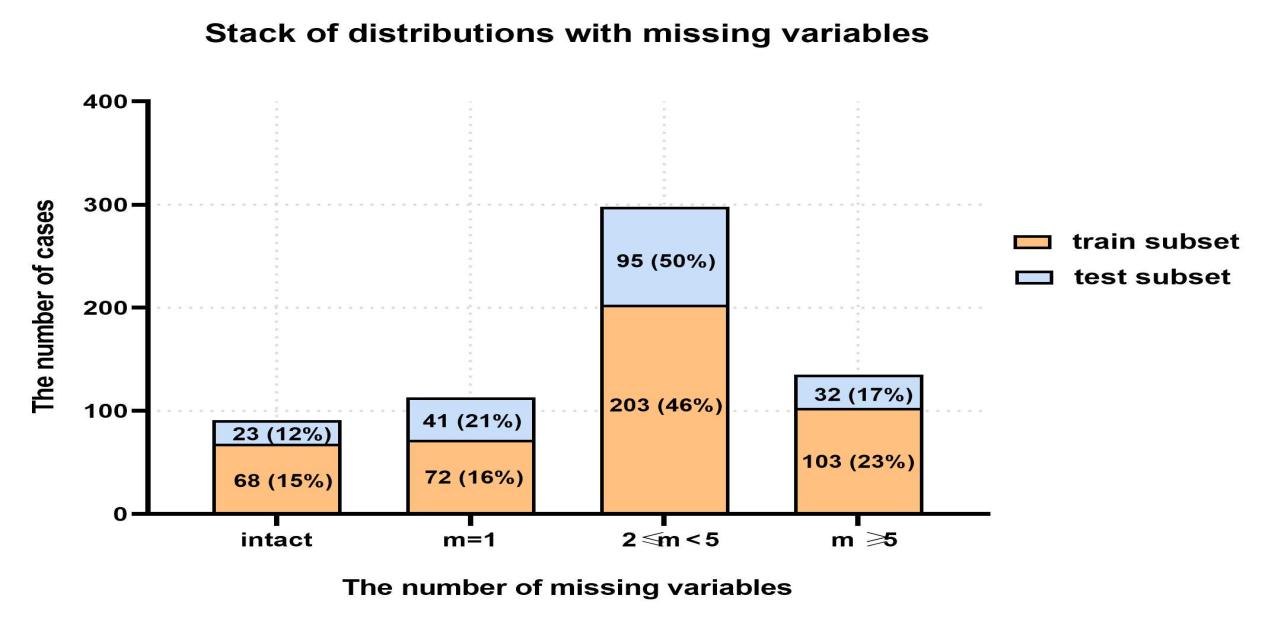


**Supplemental Figure 2**
