# Supplementary material for: Machine learning algorithms for the prediction of adverse prognosis in patients undergoing peritoneal dialysis
Source: BMC Med Inform Decis Mak. 2024 Jan 2;24:8. doi: 10.1186/s12911-023-02412-z (PMC10763100; doi:10.1186/s12911-023-02412-z)
Supplement: Supplementary file 2 — Additional file 2: Supplemental Table 1. The SHAP value of the top 20 variables. [file 12911_2023_2412_MOESM2_ESM.docx]

**Supplemental Table1.** The SHAP value of the top 20 variables

| Abbreviation of features | SHAP value |
| --- | --- |
| Age | 0.14 |
| TIBC | 0.11 |
| Prealbumin | 0.08 |
| HDL_c | 0.07 |
| Serum_albumin | 0.07 |
| FBG | 0.07 |
| Weight | 0.07 |
| SF | 0.07 |
| SBP | 0.07 |
| Vd | 0.06 |
| BMI | 0.06 |
| Education | 0.05 |
| Serum_phosphorus | 0.05 |
| IPTH | 0.05 |
| Chlorine | 0.04 |
| Admitted_date | 0.04 |
| Total cholesterol | 0.04 |
| ESR | 0.03 |
| CKMB | 0.03 |
| Creatinine | 0.03 |

TIBC: total iron binding capacity; HDL-c: high-density lipoprotein cholesterol;

FBG: fasting blood glucose; SF: serum ferritin; SBP: systolic blood pressure;

Vd: vitamin D; BMI: body mass index; IPTH: intact parathyroid hormone;

ESR: erythrocyte sedimentation rate; CKMB: creatine kinase myoglobin.
